# Dynamic changes and metabolic function of intestinal flora in patients with postoperative pneumonia after lung cancer surgery

**DOI:** 10.1371/journal.pone.0321016

**Published:** 2025-04-01

**Authors:** Meiling Wang, Weiting Jiang, Tingyu Wei, Zimeng Li, Yaxin Jiang, Pengcheng Zhou, Bizhen Chen

**Affiliations:** 1 Department of Healthcare-Associated Infection Management, The Second Affiliated Hospital of Fujian University of Traditional Chinese Medicine, Fuzhou, Fujian, China; 2 Department of Nursing, The Second Affiliated Hospital Zhejiang University School of Medicine, Hangzhou, Zhejiang, China; 3 Department of Infectious Diseases/Infection Control Center, The Third Xiangya Hospital of Central South University, Changsha, Hunan, China; State Key Laboratory for Diagnosis and Treatment of Infectious Diseases, CHINA

## Abstract

**Background:**

Postoperative pneumonia (POP) is the most prevalent postoperative complication following lung cancer surgery. It is a crucial factor that influences surgical success and the rapid recovery of patients. Studies on the gut-lung axis have suggested that changes in the structural and functional aspects of intestinal flora are implicated in the incidents and development of pulmonary infection. This study aims to reveal the dynamic changes and metabolic function of intestinal flora in lung cancer patients with POP, with the ultimate goal of providing novel insights and targets for the prevention and treatment of POP.

**Methods:**

This study includes three groups: healthy control group, lung cancer with POP group, and lung cancer without POP group. We collected stool samples from healthy individuals, preoperative and first post-infection stool samples from the POP group, and preoperative and first postoperative stool samples from the non-POP group. The hypervariable V3-V4 regions of 16S rRNA gene were sequenced using Illumina MiSeq high-throughput sequencing technology.

**Results:**

The alpha diversity index was lower in the POP group than in the healthy group, the beta diversity index was also different between the two groups (*P* <  0.05). *Eggerthella*, *Coprobacillus*, and *Peptostreptococcus* were abundant in the intestinal tracts of the POP group in preoperative and postoperative infections. There was a decrease in the abundance of beneficial genera such as *Blautia* and an increase in the abundance of pathogenic or opportunistic pathogens such as *Bacteroides.* The phosphatidylinositol signaling system abundance increased, whereas the abundance of phenazine, phenylalanine, tyrosine, and tryptophan biosyntheses was reduced in the POP group during postoperative infection.

**Conclusion:**

Patients with POP after lung cancer surgery have a distinct spectrum of intestinal flora. The intestinal flora displays a reduction in diversity and an increase in the presence of potential pathogenic bacteria, which impact metabolic functions.

## Introduction

Postoperative pneumonia (POP) is a new onset of pneumonia in surgical patients within 30 days of surgery and accounts for approximately 50% of all hospital-acquired pneumonia [1]. Currently, POP is the most common postoperative pulmonary and infectious complication in lung cancer, with an incidence ranging from 15% to 40% [2–4]. It has an insidious onset and complex etiology, with mixed bacterial infections in most cases. In addition, it is associated with a high rate of drug resistance, a long treatment period, difficulty in treatment, and a high morbidity and mortality rate. Studies have shown that pulmonary infections account for approximately 20%–60% of patients who die after lung cancer surgery [5]. Andalib et al. [6] found that the 5-year survival rate of lung cancer patients with POP was significantly lower than that of patients without POP (62.8% vs. 73.8%, respectively). POP worsens patient’ prognosis, prolongs hospital stay, consumes more medical resources, and causes enormous human, material, and economic losses to families and society [7]. Therefore, early prevention, control, and effective treatment of POP are crucial. The development of POP is a multifactorial process, and the risk factors include advanced age (≥70 years), smoking, mechanical ventilation, hyperglycemia, a combination of chronic obstructive pulmonary disease (COPD), etc., but its specific pathogenesis remains unclear [8,9].

A growing body of research suggests that the gut and lungs interact and regulate each other in both directions through microbial and immune functions, an interaction known as the “gut-lung axis” [10–12]. Many common clinical gastrointestinal diseases are often accompanied by respiratory symptoms, for example, more than 50% of patients with inflammatory bowel disease develop reduced respiratory function and impaired lung function; many lung diseases are also accompanied by gastrointestinal symptoms, e.g., more than half of patients with an acute exacerbation of COPD suffer from constipation, and patients with COPD are at a significantly higher risk of comorbidities with ulcerative colitis and Crohn’s disease [13]. Studies have confirmed the imbalance of intestinal flora in many lung diseases such as pneumonia [14], lung cancer [15], asthma [16], and COPD [17], etc. Intestinal flora has also been shown to be associated with infectious complications such as lung infections and their adverse outcomes in stroke, leukemia, and gastrointestinal surgery [18–21]. Dysbiosis of the gut microbiota is an important factor in developing numerous lung diseases, but the mechanisms involved have not been elucidated [22,23]. The migration of immune cells and bacterial metabolites are now considered to be the main underlying mechanisms of the “gut-lung axis”, in which the intestinal flora modulates the pulmonary immune response through lipopolysaccharides, short-chain fatty acids (SCFAs), and immune cells (e.g., T regulatory cells), as well as type 2 intrinsic lymphocytes, among others [24]. Intestinal flora also serve as novel biomarkers in clinical work as early predictors of postoperative infectious complications and potential targets for disease intervention [25,26].

Intestinal flora may provide a new approach to studying the pathogenesis and prevention of respiratory infections. However, limited research has investigated the relationship between lung cancer patients with POP and their intestinal flora, and it remains unclear whether intestinal flora changes during the development of POP in lung cancer patients and its relationship with POP. We analyzed the differences and functional changes of the intestinal flora in lung cancer patients with POP before and during the occurrence of POP, in lung cancer patients without POP before and after surgery, and in a healthy control group of people, to discover the potential key microorganisms in the occurrence and development of POP and to provide new ideas for the diagnosis and prevention of POP from the perspective of intestinal flora.

## Materials and methods

### Participants’ information

This is a case-control study. Patients undergoing thoracoscopic radical lung cancer surgery under general anesthesia with tracheal intubation from December 2020 to December 2021 were recruited. The inclusion criteria in this study were as follows: age 18 ~ 75 years; long-term local residency in Fuzhou, Fujian, China; regular defecation (1 ~ 3 times per day); clear consciousness; and no psychiatric disorders. Patients with other infectious diseases before surgery or besides POP after surgery, as well as a history of gastrointestinal diseases or surgery, other underlying lung diseases or surgery, other cancers, hypertension, diabetes mellitus, and other basic diseases, and those receiving antibiotics, probiotics, hormones, radiotherapy, or immunosuppressants 1 month before sampling, were excluded from the study. Based on POP development before discharge, the patients were divided into two groups: lung cancer POP and lung cancer non-POP groups. POP was diagnosed according to the 2018 diagnostic criteria of the “Expert consensus on prevention and control of postoperative pneumonia” [1]. About half an hour before surgery, all patients with lung cancer received second-generation cephalosporins as a prophylactic treatment (Cefotaxime Sodium for Injection, 2g, North China Pharmaceutical Hebei Huamin Pharmaceutical Co) Postoperative complications of other infectious diseases, postoperative application of antibiotics, unqualified stool samples, as well as missing or incorrect research data were dropped or excluded.

In addition, healthy volunteers from the physical examination center were recruited and included in the healthy control group using the same inclusion criteria as the patients. They should also have had no complaints of discomfort, a normal physical examination, normal liver and kidney function, a normal chest X-ray and other imaging examinations, and no basic diseases, such as hypertension or diabetes, no lung disorders or surgery, no gastrointestinal diseases or related surgeries, and no malignancies. Individuals who had received antibiotics, probiotics, hormones, radiotherapy, or immunosuppressants within 1 month before sampling were excluded.

### Stool sample collection

Stool samples were collected from each of the three groups at various time points. They were collected from the POP group at 1 day preoperatively and at the time of the first postoperative bowel movement after the occurrence of POP (mean 4.92 days postoperatively), and from the lung cancer non-POP group at 1 day preoperatively and at the time of the first postoperative bowel movement (mean 4 days postoperatively). After enrollment, stool samples from the healthy control group were randomly collected. All samples were collected in the hospital, divided into two portions, and transported on ice within 4 hours to the laboratory, where they were stored at − 80ºC till analyses.

### 16S rRNA gene sequencing

#### DNA extraction and polymerase chain reaction (PCR) amplification.

Total DNA was extracted using the E.Z.N.A.® soil kit (Omega Bio-tek, Norcross, GA, USA), according to the manufacturer’s instructions. DNA concentration and purity were determined using a NanoDrop2000 UV-vis spectrophotometer (Thermo Fisher Scientific, Wilmington, DE, USA), and DNA quality was checked by 1% agarose gel electrophoresis. The bacterial 16S rRNA V3–V4 hypervariable regions were amplified using the following primers: 338F (5′-ACTCCTACGGGAGGCAGCAG-3′) and 806R (5′-GGACTACHVGGGTWTCTAAT-3′). PCRs were performed in triplicate and comprised a 20 μL mixture containing 4 μL of 5× FastPfu Buffer, 2 μL of 2.5 mM dNTPs, 0.8 μL of each primer (5 μM), 0.4 μL of FastPfu Polymerase, and 10 ng of template DNA. They were conducted on a thermocycler PCR system (GeneAmp 9700, ABI, USA) with the following thermal profile: denaturation for 3 minutes at 95°C, followed by 27 cycles of denaturation for 30 seconds at 95°C, annealing for 30 seconds at 55°C, and extension for 45 seconds at 72°C, and a final extension at 72°C for 10 min.

#### Library construction and Illumina MiSeq sequencing.

PCR products were checked for correct size on a 2% agarose gel electrophoresis, purified using the AxyPrep DNA gel extraction kit (Axygen Biosciences, Union City, CA, USA), eluted with Tris–HCl, and was measured with Quantifluor-ST (Promega, USA) and quantified according to Illumina Miseq platform (Illumina,San Diego,USA) Standard Operating Procedures for generating sequencing libraries from the purified amplified fragments: (1) Connect the Y-shaped connector; (2) the use of magnetic bead screening to remove the joint self-connecting segment; (3) enrichment of library templates by PCR amplification; (4) NaOH denatured to produce single-stranded DNA fragments. At last, purified amplicons were pooled in equimolar concentrations and paired-end sequenced (2 ×  300) on an Illumina MiSeq platform (Illumina, San Diego, CA, USA) according to the standard protocols.

#### Bioinformatics and statistical analyses.

All bioinformatics analyses and plots were available on the website at https://www.bioincloud.tech/. The analysis was conducted using the “Atacama soil microbiome tutorial” of QIIME2 docs along with customized program scripts (https://docs.qiime2.org/2019.1/). Using the QIIME tools import program, raw data FASTQ files were imported into the format that could be operated by the QIIME2 system. The demultiplexed sequences from each sample were quality filtered and trimmed, denoised, merged, and then the chimeric sequences were identified and removed using the QIIME2 dada2 plugin to generate the feature table of the amplicon sequence variant (ASV). The QIIME2 feature-classifier plugin was then used to generate the taxonomy table by aligning ASV sequences to a pretrained GREENGENES 13_8 99% database (trimmed to the V3 ~ V4 region bound by the 338F/806R primer pair). The diversity metrics were calculated using the core-diversity plugin within QIIME2. A linear discriminant analysis effect size (LEfSe) was calculated to determine biomarkers between groups. The KEGG metabolic pathway of the bacterial population was predicted using the Phylogenetic Investigation of Communities by Reconstruction of Unobserved States (PICRUSt) software, and analysis of variance (ANOVA) Duncan analysis was used to find the significant difference pathway, combined with grouping information.

For baseline data and α-diversity indices, quantitative data were expressed as the mean ±  standard deviation (x̄ ± s). For quantitative data that conformed to a normal distribution, comparisons between two groups were made using the independent samples t-test. Non-normally distributed quantitative data were compared using the rank sum test, with the Mann-Whitney U test employed for comparisons between two groups and the Kruskal-Wallis test for comparisons among three groups. Categorical data were expressed as frequencies (%), and comparisons were made using the chi-square test. The Statistical Package for the Social Sciences software, version 26.0, was used for statistical analyses. *P* values of <  0.05 were considered statistically significant.

#### Ethics approval and informed consent.

This study was approved by the Ethics Committee of the Second Affiliated Hospital of Fujian University of Traditional Chinese Medicine (no. 2018-KL014), and all participants signed written informed consent.

## Results

### Characteristics of the studied cohort

**Fig 1:**
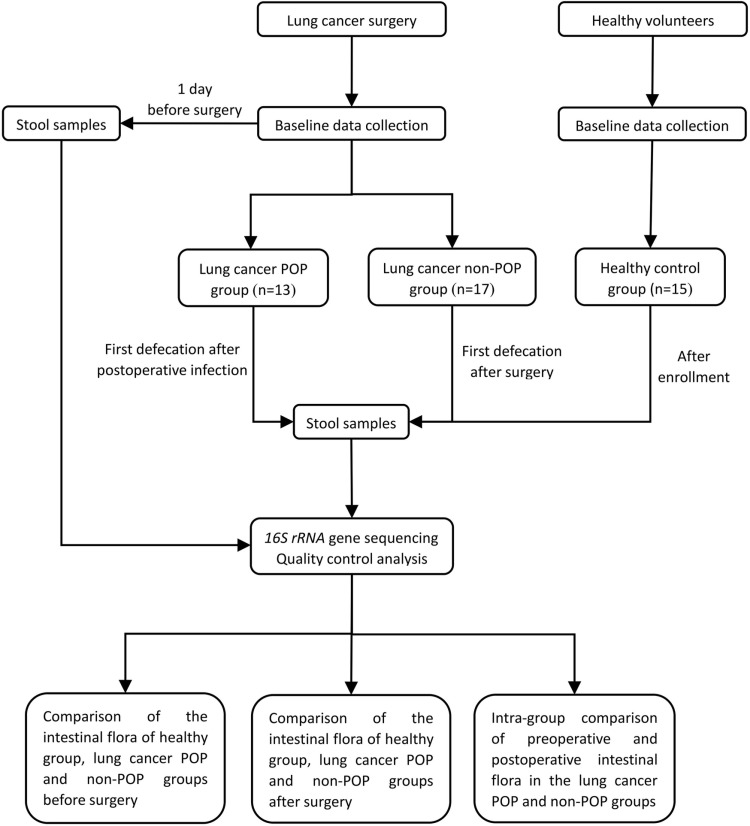
Study design and flow diagram.

45 subjects were finally included in the study: 15 healthy controls, 13 lung cancer patients with POP, and 17 lung cancer patients without POP (Fig 1). There was no statistically significant difference in age, gender, body mass index, or smoking status among the three groups, and no statistically significant difference in the duration of surgery, duration of anesthesia, and preoperative serum albumin between the POP and non-POP groups (*P* >  0.05) (Table 1). The symptoms of pneumonia appeared in 13 POP patients within 3 to 5 days after surgery, two patients were sent for sputum culture and obtained positive results of *Staphylococcus aureus*. All patients with lung cancer surgery recovered and were discharged from the hospital without any deterioration, unexpected return to the ICU, or death.

**Table 1:** Baseline characteristics of participants.

		Healthy controls (n = 15)	POP group (n = 13)	Non-POP group (n = 17)	χ^*2*^*/ Z/ t*	*P*
Age (year)		53.47 ± 7.30	56.00 ± 14.58	48.06 ± 11.72	4.132^a^	0.127
Gender	Male	5 (33.33%)	7 (53.85%)	4 (23.53%)	3.003^b^	0.223
	Female	10 (66.67%)	6 (46.15%)	13 (76.47%)		
BMI (Kg/m^2^)		23.95 ± 3.73	22.91 ± 3.23	21.71 ± 2.47	1.929^a^	0.381
Smoker	Yes	4 (26.67%)	5 (38.47%)	2 (11.76%)	2.903^b^	0.234
	No	11 (73.33%)	8 (53.33%)	15 (88.24%)		
Pathological type	Squamous carcinoma	/	2 (15.38%)	0 (0%)	2.802^b^	0.094
	Adenocarc-inoma	/	11 (84.62%)	17 (100%)		
Cancer Stage	Stage I	/	10 (76.92%)	14 (82.35%)	0.814^b^	0.665
	Stage II	/	1 (7.69%)	2 (11.76%)		
	Stage III	/	2 (15.38%)	1 (5.88%)		
Preoperative serum albumin (g/L)			40.32 ± 3.72	41.99 ± 2.97	-1.369^c^	0.182
Length of surgery (min)			147.77 ± 73.95	146.76 ± 58.67	0.042^c^	0.967
Length of anesthesia (min)			188.15 ± 75.63	183.65 ± 60.03	0.182^c^	0.857

^a^: Rank sum test;

^b^: χ2 test;

^c^:t-test.

### Alpha diversity analyses

**Fig 2:**
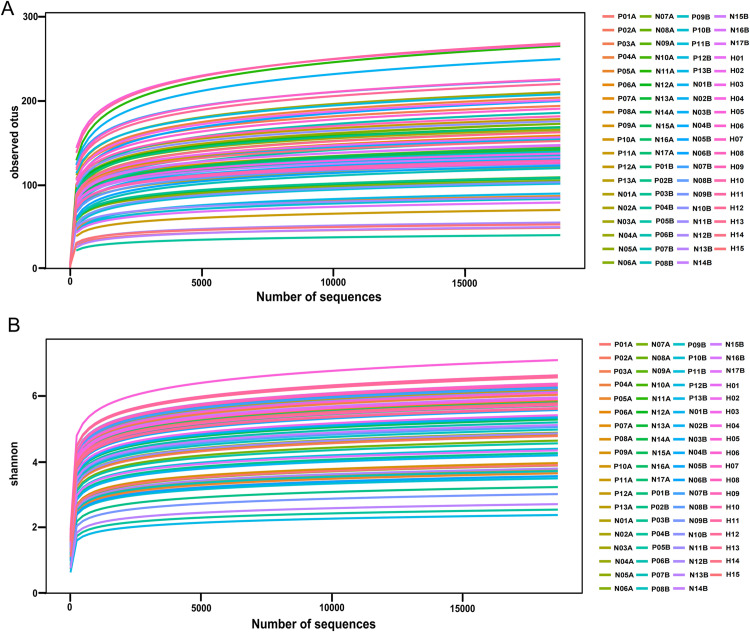
ASV rarefaction curves and Shannon index curves. (A) ASV rarefaction curves; (B) Shannon index curves.

As Good’s coverage of all specimens reached 99.99%, the sequencing results could reflect the actual population composition. The ASV rarefaction curves (Fig 2A) and Shannon’s index curves (Fig 2B) observed gradually leveled out and hit a plateau, indicating that the present sequencing results were sufficient to reflect the diversity of the samples, and the sequencing volume and depth were adequate.

**Fig 3:**
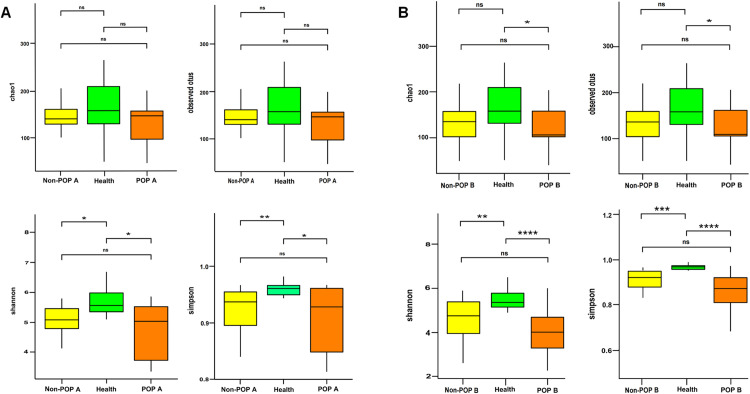
Bacterial alpha diversity. Chao1 index and Observed species index reflect species richness; the Shannon index and Simpson index reflect species diversity and evenness. Wilcox Test was used to compare the differences. (A) The comparison of intestinal flora Alpha diversity between healthy, POP and non-POP groups before surgery. (B) The comparison of intestinal flora Alpha diversity between healthy group, POP group after postoperative infection and non-POP group after surgery. * *P* < 0.05, **P < 0.01, ***P < 0.001, ****P < 0.0001.

First, the overall characteristics of intestinal flora in the preoperative stool samples of the healthy, POP, and non-POP groups were described and compared (Fig 3A) to determine whether the preoperative intestinal flora of patients with POP varied and differed when POP did not occur. The results showed that Shannon’s index and Simpson’s index of the POP group and non-POP group were lower than those of the healthy group (*P* <  0.05). There was no significant difference in chao1 index among the three groups, while there was with no statistical difference in the preoperative intestinal flora alpha diversity index between the POP and non-POP groups (*P* >  0.05). To further understand the association between intestinal flora and POP development and progression after lung cancer surgery, the intestinal flora in the POP group after postoperative infection, the non-POP group after surgery, and the healthy group were compared (Fig 3B). The postoperative alpha diversity index in the POP group was significantly lower than that in the healthy group (*P* <  0.05), with no statistical difference between the POP and non-POP groups (*P* >  0.05).

### Beta diversity analyses

**Fig 4:**
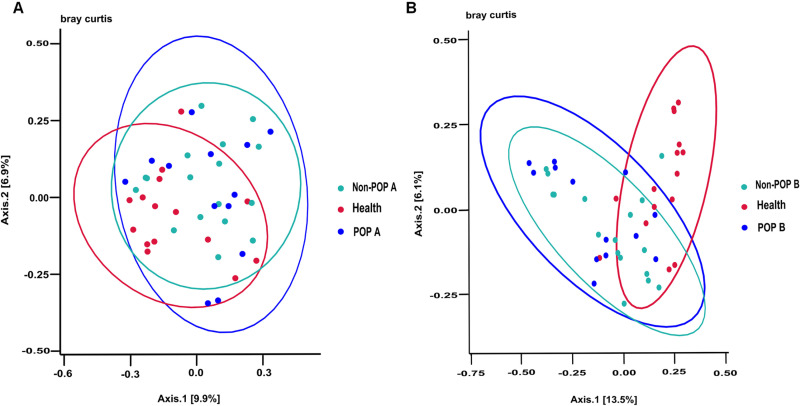
Bacterial beta diversity. Bray-Curtis distance PCA plots. The Bray-Curtis distance matrices were used to calculate the Beta diversity, indicating the difference in bacterial community structure. The PERMANOVA test was used to compare the differences. (A) The comparison of intestinal flora Beta diversity between healthy, POP and non-POP groups before surgery. (B) The comparison of intestinal flora Beta diversity between healthy group, POP group after postoperative infection and non-POP group after surgery. Each point represents a sample, and the points of different colors belong to different groups. The closer the distance between two points, the higher the similarity of microbial community structure between two samples and the smaller the difference.

The distance between points in the Bray-Curtis distance principal component analysis plots illustrates the diversity and similarity of samples between groups (Fig 4). The POP and non-POP groups had closer distances between points in each sample, confounding crossover, and weak grouping effects, but the healthy group demonstrated relatively different aggregation characteristics. The permutational multivariate analysis of variance (PERMANOVA) test results revealed significant differences between the POP and healthy groups both preoperatively and during POP occurrence (*P* = 0.036, 0.001 < 0.05), with no significant difference with the non-POP group (*P* = 0.670, 0.437 > 0.05).

### Bacterial composition and variation analyses

**Fig 5:**
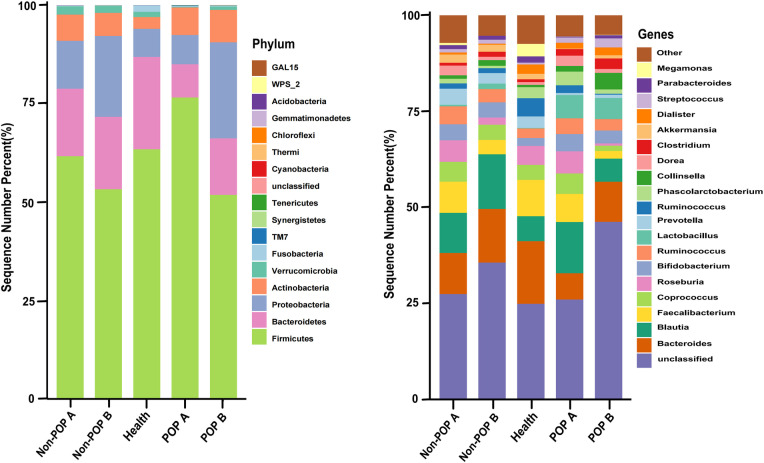
Relative distribution of species bar chart. Only the top 20 species in relative abundance were shown. Other species with low relative abundance were classified as “Other”.

*Firmicutes*, *Bacteroidetes*, *Proteobacteria*, and *Actinobacteria* were the most dominant phyla in lung cancer patients with POP accounting for more than 95% of all cases (Fig 5). The Kruskal-Wallis test is used to analyze differences in species abundance. When the healthy and two lung cancer groups were compared preoperatively and postoperatively, there were no significant differences at the phylum level. However, at the genus level, preoperatively, the abundance of *Veillonella* (*P* = 0.044) and *Eggerthella* (*P* = 0.045) was greater in the POP group than in the healthy and non-POP groups, and postoperatively, the abundance of *Eggerthella* (*P* = 0.023) was greater during the occurrence of POP in the POP group than in the healthy and non-POP groups.

**Fig 6:**
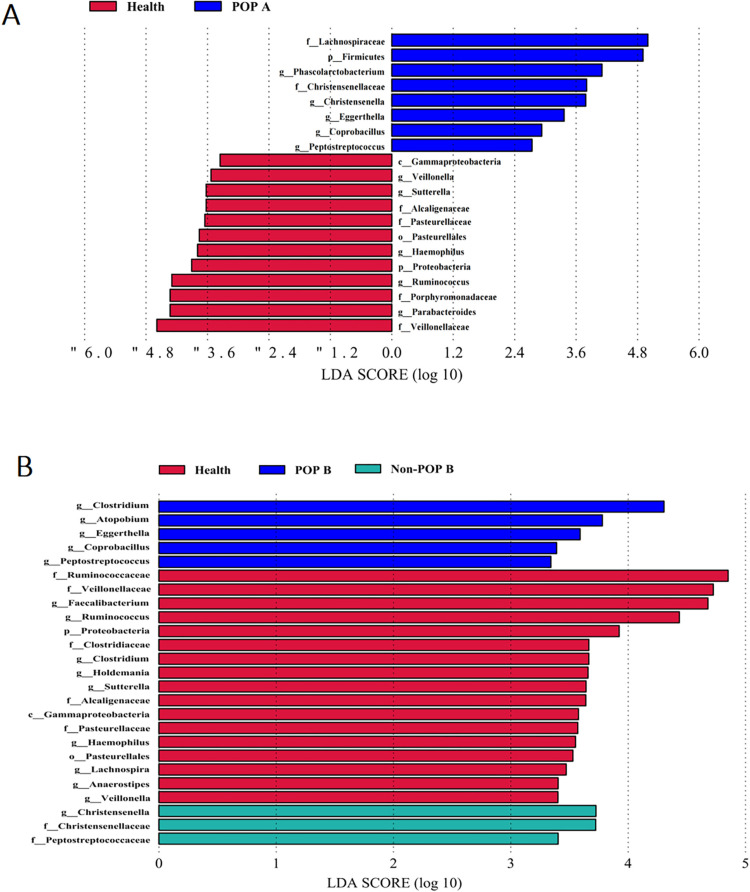
Bacterial LEfSe analysis. (A) The comparison of intestinal flora LEfSe analysis between healthy, POP and non-POP groups before surgery. (B) The comparison of intestinal flora LEfSe analysis between healthy group, POP group after postoperative infection and non-POP group after surgery. Different colors represent different groups, and each horizontal column represents a significantly different species in the group. The length of the column corresponds to the LDA score, and the higher the LDA score, the greater the difference. Only taxa achieving an LDA significant threshold >  2 were shown.

The preoperative LEfSe analysis results of the POP, non-POP, and healthy groups revealed 8 significantly distinct species in the POP group, 12 in the healthy group, and no characteristic dominant flora in the non-POP group (Fig 6A). The results of LEfSe analysis in the POP group after postoperative infection, the non-POP group postoperatively, and the healthy group revealed 5 significantly distinct species in the POP group, 17 in the healthy group, and 3 in the non-POP group (Fig 6B). In the POP group, *Eggerthella*, *Coprobacillus*, and *Peptostreptococcus* were abundant in the intestine during both preoperative and postoperative infection.

After postoperative infection in the POP group, *Firmicutes* were less abundant, but *Proteobacteria* were more abundant than in the preoperative period (*P* < 0.05). The abundance of beneficial genera, such as *Blautia*, *Lactobacillus*, *Faecalibacterium*, *Bifidobacterium*, and *Coprococcus*, decreased, whereas the abundance of pathogenic bacteria, such as *Bacteroides* and *Collinsella*, or opportunistic pathogenic bacteria increased (Table 2).

**Table 2:** Changes in relative abundance of intestinal flora in POP group.

Species	Preoperative relative abundance %	Postoperative relative abundance %	Variation trend	*P*
Phylum				
*Firmicutes*	76.58	51.87	↓	0.020
*Bacteroidetes*	8.44	14.35	↑	0.522
*Proteobacteria*	7.46	24.37	↑	0.033
*Actinobacteria*	6.98	8.25	↑	0.817
Genera				
*Blautia*	13.34	5.98	↓	0.026
*Faecalibacterium*	7.32	2.02	↓	0.079
*Bacteroides*	6.85	10.46	↑	0.457
*Lactobacillus*	6.09	5.43	↓	0.575
*Roseburia*	5.72	0.63	↓	0.008
*Coprococcus*	5.34	1.34	↓	0.106
*Bifidobacterium*	4.53	3.33	↓	0.817
*Ruminococcus*	4.08	3.01	↓	0.024
*Phascolarctobacterium*	3.54	1.14	↓	0.936
*Collinsella*	1.49	4.27	↑	0.883

The upper arrow indicates an increase in postoperative relative abundance compared to the preoperative, whereas the lower arrow indicates a decrease.

### Functional prediction of intestinal bacteria

**Fig 7:**
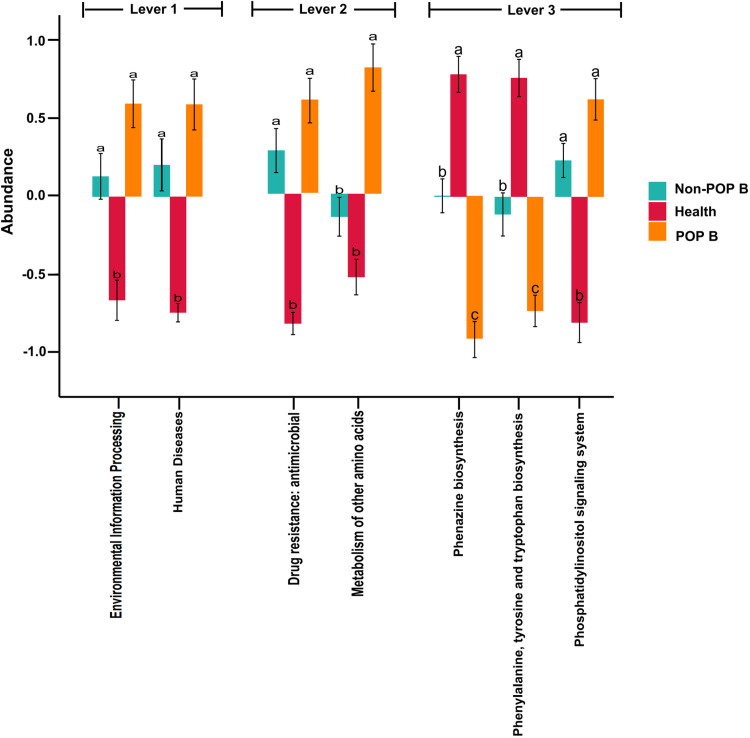
Bacterial differential metabolic pathways. The horizontal coordinates are pathway names, different colors of each pathway indicate different subgroups, and the same letter above two subgroups means the difference is not significant, otherwise the difference is significant. The vertical coordinates represent the abundance level, positive values represent relative abundance greater than the mean, negative values represent relative abundance less than the mean.

PICRUSt was used to predict the function of the intestinal flora of the three groups and was paired with grouping information for analyses. We filtered the pathways observed in less than 50% of the total samples. The results revealed that the functional composition of the three groups was typically similar before surgery, with a mild, non-significant difference detected between the groups. However, a postoperative analysis of the three groups revealed seven significantly different pathways (Fig 7). The phosphatidylinositol signaling system abundance increased,whereas the biosynthesesof phenazine, phenylalanine, tyrosine, and tryptophan were reduced in the lung cancer POP group during postoperative infection.

## Discussion

Although POP is common in lung cancer and has serious consequences, its pathogenesis remains unclear. Our results showed that both at the time of preoperative and postoperative infections, the alpha diversity index of the intestinal flora was significantly lower in lung cancer patients with POP compared with the healthy population; their community structure was altered, characterized by low diversity and low abundance of key commensal bacteria. There were no statistically significant differences in alpha and beta diversity between POP and non-POP patients (*P* >  0.05), but LEfSe analyses identified the respective differential species between the two groups, indicating that the intestinal flora of POP and non-POP patients still have distinct characteristics.

The results of species composition and differential analysis showed that the composition of the intestinal bacterial profiles of the three groups was generally the consistent, but differed in specific compositional proportions. The abundance of *Eggerthella* was simultaneously increased in the intestinal flora of the POP group preoperatively and during the occurrence of POP; and LEfSe analysis showed that *Eggerthella*, *Peptostreptococcus*, and *Coprobacillus* were reflected in high abundance in the intestinal flora of POP patients both preoperatively and at the time of POP onset. *Eggerthella* is a kind of proinflammatory bacteria, as a new pathogen, associated with bacteremia, abdominal infection and other infectious diseases, a retrospective cohort studies found that they can cause impaired host immunity and lead to opportunistic infections [27–29]. Charlson et al noticed that an increase in *Proteobacteria* such as *Eggerthella,* as well as exposure to cigarette smoke induced intestinal microecological disturbances and functional changes in the intestinal mucosal barrier, exacerbating pulmonary inflammatory responses in elderly patients with COPD [30]. *Peptostreptococci* can also cause infections in various tissues and organs of the human body in clinical practice, with mixed infections in the respiratory tract, lung pleura, and other parts of the body being the most common [31,32]. Studies have shown that *Peptostreptococci* is prevalent in the microbiota of some cancerous tissues and can inhibit immunity through the interaction of their surface proteins with the epithelial cell receptor integrins α2/β1, which promotes cell proliferation and a pro-inflammatory immune microenvironment. *Coprobacillus* bacteremia–associated bacteria and their potential secondary infections are more likely to cause severe host disease [33,34]. A recent study showed that the abundance of intestinal *Coprobacillus*, *Clostridium ramosum*, and *Clostridium hathewayi* in patients with coronavirus disease 2019 correlated with disease severity [35]. *Eggerthella*, *Coprobacillus*, and *Peptostreptococcus* are all infection- and inflammation-associated bacteria and are enriched in the intestines before and after the onset of POP in patients with lung cancer. We hypothesize that the differences and variations in the above genera may play an important role in the development of POP, and have the potential to be microbial markers for precise prevention and control of POP in future clinical work.

We also performed a predictive analysis of the functional composition of intestinal flora species in each group. The abundance of the phosphatidylinositol signaling system in intestinal flora was increased in lung cancer patients with POP. Phosphatidylinositol 3-kinase (PI3K) signaling has been shown to drive a variety of cellular responses, including proliferation, migration, growth, and survival, and can be overactivated in pathological states, such as cancer [36]. The δ isoform is predominantly found in leukocytes and regulates immune cell function, which can be activated in response to immune stimulation. The metabolism of other amino acids was increased in lung cancer patients with POP, whereas the biosynthesis of phenazine, phenylalanine, tyrosine, and tryptophan biosyntheses was significantly lower than in non-POP patients and healthy individuals. Phenazines and their derivatives have shown promising biological activities in various fields, including antibacterial, antiparasitic, neuroprotective, anti-inflammatory, and anticancer properties [37]. Aromatic amino acids, such as phenylalanine, tyrosine, and tryptophan, are basic components of proteins that can be metabolized by both the organism and the intestinal flora. In addition, aromatic amino acids and their derivatives play important roles in organism immunity and neuronal response, as well as anti-inflammatory, antiviral, and anticancer properties [38,39]. We hypothesize that in the presence of lung cancer and infection, the intestinal microecology and microenvironment changed, nutrient metabolism increased, relevant bacterially regulated signal transduction pathways are altered, and bacterial biosynthesis and raw material supply are decreased.

We compared the intestinal flora of lung cancer patients before and during the occurrence of POP, and it showed that the percentage of *Firmicute*s decreased during the occurrence of POP, and the percentage of *Proteobacteria* increased. Studies have shown that a decrease in the *Firmicute*s leads to a decrease in the synthesis of short-chain fatty acids (SCFAs), butyric acid, etc., which may affect intestinal metabolism and mucosal immunity, causing obstacles in pathogenic bacteria clearance and systemic immune-inflammatory reactions, and promoting the development of diseases [40,41]; whereas *Proteobacteria* as potential identifiers of microecological dysbiosis and disease, and its increase in certain intestinal environments may lead to local or systemic inflammation and metabolic dysfunction, which may contribute to the development of the disease [42]. At the genus level, the abundance of beneficial genera, such as *Blautia*, *Lactobacillus*, *Faecalibacterium*, and *Bifidobacterium*, decreased in patients with POP, while the abundance of pathogenic or opportunistic pathogenic bacteria, such as *Bacteroides*, increased. Studies of the gut-lung axis have shown that the intestine and the lung are physiologically and functionally interconnected, and pathologically interact cross-talk. The development of POP in lung cancer patients and the disturbance of intestinal flora are also be interactive and interrelated. Intestinal microbiology plays a remote regulatory role in the lungs through flora shift and immune response effects. In the lung disease state, patients have an imbalance in the intestinal microecology, with a decrease in the number of beneficial bacterial species, growth or population shifts of conditionally pathogenic bacteria, and there is an alteration in the metabolic profile of the host, with a decrease in beneficial metabolites and/or an increase in pro-inflammatory metabolites, which results in a compromised intestinal barrier, an increase in intestinal permeability, bacterial translocation, an activation of intestinal immunity, and an increase in immune cell interactions. At the same time, a large amount of intestinal endotoxin and inflammatory factors are released and enter the lungs through the bloodstream and mesenteric lymphatic vessels, which leads to the aggregation of inflammatory cells, such as neutrophils and macrophages, and the initiation of pulmonary inflammatory cascade, thus inducing the occurrence of lung infection and injury.

Patients undergoing lung cancer surgery now have shorter preoperative hospital stays and less specific treatment with various preoperative tests, thanks to the development of fast-track surgery. We collected stool samples from patients undergoing lung cancer surgery 1 day before surgery to ensure that they accurately reflect the characteristics of the intestinal flora. However, the study also has certain limitations. First, a small number of patients, and the limited sample size of this study makes it difficult to analyze possible associations between the other variables with POP. Meanwhile, we lack data on clinical bacteriology, various factors in the perioperative period, such as surgical trauma, psychological stress, and medication, etc., may affect the final result presentation to some extent. The results of this study suggest an association between POP and intestinal flora, but the causal relationship needs to be explored in depth. Intestinal flora plays an important role in immunity and metabolism. However, our analysis focused only on the characteristics and variations of intestinal flora composition and did not extensively investigate the correlation between the intestinal flora, systemic immune-related indices, and metabolic functions. Further experiments on their specific mechanism of action are still needed in the future.

## Conclusion

In conclusion, the intestinal flora of lung cancer patients with POP was further disrupted, with reduced intestinal flora diversity, significant differences in flora structure, decreased beneficial bacteria, and increased pathogenic or opportunistic pathogenic bacteria. *Eggerthella*, *Peptostreptococcus*, and *Coprobacillus* were significantly enriched in lung cancer patients with POP and were related to the development of POP in lung cancer. The change of intestinal microflora also affected the metabolic function. At the onset of POP in lung cancer, the abundance of the phosphatidylinositol signaling system increased, whereas phenazine, phenylalanine, tyrosine, and tryptophan biosyntheses decreased.
